# An effective-charge model for the trapping of impurities of fluids in channels with nanostructured walls

**DOI:** 10.1186/1556-276X-8-19

**Published:** 2013-01-10

**Authors:** Manuel V Ramallo

**Affiliations:** 1 Departamento de Física da Materia Condensada, Universidade de Santiago de Compostela, Santiago de Compostela, 15782, Spain

**Keywords:** Nanostructures embedded in larger systems, Fluid impurity trapping in nanostructures, Effective-charge density

## Abstract

We present model equations for the trapping and accumulation of particles in a cylindrical channel with nanostructured inner walls when a fluid passes through, carrying a moderate load of impurities. The basic ingredient of the model is the introduction of a phenomenological ‘effective-charge density’ of the walls, related to the electrical charges exposed in the nanotexture. The effective charge is gradually reduced as the flow runs through the channel and the trapped impurities cover the internal walls. Based on the proposed equations, the position and time dependence of the areal density of trapped impurities, and the filtration performance, may be calculated. It is proposed that experimentally testing these results may help to understand the enhanced trapping capability observed in many diverse nanotextured channel structures.

## Background

Recently, [1-8] researchers from both academia and industry have experimentally demonstrated that a variety of nanostructures and nanotextured media (see some examples later in this introduction) can efficaciously trap nanoimpurities carried by fluids when a flow is induced by external hydrostatic pressure. These findings are not only scientifically interesting, but also promising for the socially and economically important application of purification of drinking water and other liquids [4,7-9]. When compared to conventional porous filters, the new media have the important advantages of retaining impurities of sizes typically in the tens of nanometers and, at the same time, presenting a resistance to hydrodynamic flow orders of magnitude smaller than what conventional models would predict for channels of diameters as small as the particles being trapped.

Roughly, we can divide the structures presenting such enhanced impurity trapping capability into two groups: (a) The first group corresponds to those formed by nanometric-diameter channels through which the fluid flows [1-4]. A well-known example is the nanotube arrays grown and experimentally tested by Srivastava and coworkers [1]. Other specially interesting examples are graphene membranes although, by now, they have been probed only through molecular dynamics simulations [2]. In any nanometric-diameter channel, simple size exclusion will play a major role in the retention of nanoimpurities. However, in addition, these structures also exhibit remarkable capability to trap some ions significantly smaller than the channels’ diameter [1,2]. The resistance to flow is observed to be well lower than what conventional models predict for these diameters, a phenomena often attributed to water-nanostructure interactions (see, e.g., [1]) though not yet fully understood at the quantitative calculation level. (b) The second group corresponds to nanostructures embedded in larger structures, resulting in filters composed by channels with micrometric diameters and inner walls coated with nanoparticles. Examples are conventional microfilters coated with Y_2_O_3_[5], ZrO_2_[6], or Al_2_O_3_[7,8] nanopowders (further examples can be found in the reviews [3,4,9]). These structures have been observed by their growers to have a surprisingly good filtration performance for nanometric impurities, as small as approximately 10 nm, in spite of the relatively large diameter of the channels (note that in a channel with a diameter of 1 *μ*m only about 0.04% of the fluid will transit closer than 10 nm from the walls) [3-9]. Their hydrodynamic resistance is quite low, similar to the one of conventional micrometric filters. Their trapping capability is observed to depend on pH and zeta potential [5-8] and, thus, electrostatic and polar attraction may be suspected to play a significant role in the filtration mechanism and dynamics. However, attempts to modelize them have been scarce. The authors of [7,8] empirically characterized their filters using general-purpose plug-flow adsorption models, like those used for column chromatography, and fitting the Langmuir and BET isotherms. While these models may be very useful for the design of improved filters, further theoretical work modelling the trapping capability and time evolution explicitly in terms of the charge of the walls, and also more detailed measurements, seems to be needed to further understand the impurity trapping mechanism and dynamics of this type of channels with nanostructured walls.

The purpose of this ‘Nano Idea Letter’ is to propose a specific model for the nanoimpurity trapping capability of cylindrical-like channels with nanostructured inner walls of the type composing filters of category ‘b’ in the previous paragraph. We explore theoretically a simplified but realistic view in which the improved filtration capability is primarily due to the fact that the nanotexturing exposes electrical charges in the walls which induce both electrostatic and van der Waals attractions over the impurities in the fluid. This nanostructuring also provides chemical anchors for the binding of those impurities once they collide with the channel walls. Correspondingly, our basic ingredients will be the introduction of an effective-charge density, *z*_*e*_, of the inner walls of the channels and writing down as a function of *z*_*e*_ the impurity trapping probability. As it could be expected, *z*_*e *_will depend on the areal density *n* of impurities already trapped in the inner walls of the channel. We obtain within the model the evolution of *n* and *z*_*e *_with position *x* and with time *t* when the liquid is flowing through the channel.

The model produces agreement with the initial trapping performances quantitatively reported by experimentalists in various systems. Also, we propose that further detailed measurements as a function of time may be crucial to test these ideas more thoroughly. We believe that some aspects of the model could also be useful to partly explain the trapping of the smaller ions in the nanodiameter channels of category ‘a’. However, its full applicability to that case is limited by our use of classical dynamics for the carrying fluid. Hence, we do not focus here on that category (also, for these nanodiameter channels, in which the number of fluid atoms is manageably small, molecular dynamics simulations as those in [2] could be a more reliable, albeit not general, approach).

## Obtainment of an equation for the areal density of trapped impurities in a channel with nanostructured walls

### Initial modelling and notations

**Figure 1:**
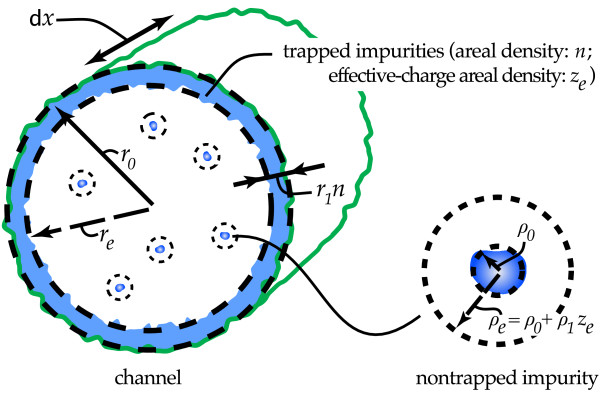
**Representation of a nanostructured channel filter as modelled in the present letter.** The nominal shape of the channel is supposed to be cylindrical with length *L*, and the figure shows only the differential slice with axial coordinate from *x* to *x* + d*x*. The radiuses *r*_0 _and *ρ*_0 _correspond to the average dimensions of the bare channel and impurities. The effective radiuses *r*_e _and *ρ*_e _vary as trapped impurities cover the inner wall, via their dependences on, respectively, the areal density *n* of trapped impurities and on the areal density *z*_e _of effective charge of the inner wall. This *z*_e _reflects that exposed charges in a nanostructured surface attract the impurities in the fluid and also constitute binding anchors for those impurities. It is expected to diminish as impurities cover the surface, for which we assume the simple *z*_e_(*n*) dependence given by Equation 1 of the main text.

Our starting point, and most of our basic notations, is illustrated in Figure 1. We consider a channel with nanostructured inner walls, its nominal shape being cylindrical-like with average radius *r*_0_and length *L*. We divide it into slices along the axial coordinate *x*, each with differential thickness *dx*. A fluid flows through the channel due to externally applied hydrostatic pressure, carrying a load of impurities. Some of those impurities will become trapped by the inner wall of the channel, then reducing its effective radius to a value *r*_*e*_(*x*,*t*) =* r*_0_−*r*_1_*n*(*x*,*t*), where *t* is the time, *n* is the number areal density of trapped impurities in the inner wall, and *r*_1_is a constant proportional to the average volume of impurities. Throughout this letter, by ‘areal density’ we refer to quantities normalized using the nominal area of the inner wall (2*Πr*_0_*dx* for a differential slice) and not the cross section of the channel. Also, for simplicity, we consider all impurities equal among them (subsequent generalization to multiple chemical species should be easy). The average radius of the impurities is noted *ρ*_0_. The impurity concentration in the fluid is considered to be moderate enough as to not significantly affect its viscosity and as for the impurities in the fluid to be noninteracting with each other (specially when colliding with the channel wall).

### Effective-charge density of the inner wall, *z*_*e*_

We now introduce the important concept of a phenomenological ‘effective charge’ of the inner wall of the channel. We quantify this effective charge via its areal density *z*_*e*_, and as already commented on in the introduction, it reflects the fact that the nanostructured walls expose charges that induce both electrostatic and van der Waals attractions over the components of the impurities in the fluid. Indeed, *z*_*e*_ will depend on the areal density of already trapped impurities *n* (which will screen out the wall) and also on the chemistry specifics of the wall and impurities. Let us focus on the mutual interplays between *n* and *z*_*e*_ and in obtaining an equation for their evolution with time as flow passes through the channel. In particular, the interdependence *z*_*e*_(*n*) may be naturally expected to be continuously decreasing when *n* increases, to take a finite value *z*_0_ at *n *= 0 (clean filter), and to saturate to zero when *n* reaches some critical value *n*^sat^ at which all active centers of the wall become well covered by impurities. We thus postulate the simplest *z*_*e*_(*n*) dependence fulfilling such conditions:

(1) $$\begin{array}{lll} \;z_{e}\;\; & =\;\;\left\{\begin{array}{ll} z_{0}\left(1-\frac{n}{n^{\text{sat}}}\right), & \;\text{if}\;n<n^{\text{sat}}\\ 0, & \;\text{if}\;n\geq n^{\text{sat}} \end{array}\right)\; & \;\\ \;\; & =\;\;z_{0}\left(1-∥)\frac{n}{n^{\text{sat}}}∥)\right),\; \end{array}$$

where the notation ∥…∥ stands for min{1,…}. Obviously, other sensible choices for *z*_*e*_(*n*) are possible such as, e.g., *z*_*e*_(*n*) =* z*_0_(1−∥*n*/*n*^sat^∥)*z*_1_, with *z*_1_ as a positive coefficient that probably covers at a good approximation most actual possibilities depending on its value, as it corresponds to a *z*_*e*_(*n*) functionality with downwards curvature if 0 <* z*_1 _< 1, to no curvature for *z*_1 _= 1 (i.e., Equation 1), and to upwards curvature for *z*_1 _ >1. For simplicity, we shall consider in this letter only the case *z*_1 _= 1. Note also that *z*_*e*_ may depend on position and time via the *n*(*x*,*t*) dependence.

### Impurity trapping probabilities as a function of ***z***_e_and ***n***

The role played by *z*_*e *_in our model will be in fact twofold. First, it affects how large the distance is within which if the impurity approximates the inner wall then the latter attracts the former so much as to consider it as a collision. This attraction distance may be seen as an effective radius, *ρ*_*e*_, of the impurity (see Figure 1), so if the distance from the center of the impurity to the center of the channel is larger than *r*_*e*_−*ρ*_*e*_, the impurity will actually touch the wall (dressed with already trapped impurities). Let us discuss the *ρ*_*e*_(*z*_*e*_) dependence. We consider first the simplest case of an unscreened electrostatic interaction, in which the potential energy of an impurity at a distance *ρ*_*e*_ from the wall is $\propto z_{e}/\rho_{e}$. Its kinetic energy associated to the thermal agitation is $\propto k_{\text{B}}T$. By equating both and also taking into account the finite bare size of impurities, we obtain $\rho_{e}=\rho_{0}+\rho_{1}^{\prime }z_{e}$ as a reasonable approximation, where $\rho_{1}^{\prime }$ is a constant inversely proportional to temperature. More interesting is the case in which ions in the carrying fluid partly screen out the electrostatic interaction. The precise algebraic distance dependence of the screened electrostatic energy may be different for each specific channel, fluid, and impurity, but we adopt here the common Debye-Hückel approximation in which this energy at a distance *ρ*_*e*_ from the surface is taken as $\propto (z_{e}/\rho_{e})\exp(-\rho_{e}/\lambda_{\text{D}})$ where *λ*_D_ is the so-called Debye length. In aqueous liquids, *λ*_D _is a function of the ionic strength, and for concreteness, we will consider it to be dominated by the background electrolytes in the fluid rather than by the impurities to be filtered out (this seems to be the case at least of the measurements in [5,6]), so *λ*_D_ is essentially independent on the concentration of the impurities to be trapped by the channel walls. By equating now the screened potential energy at *ρ*_*e*_ to the thermal kinetic energy, we get

(2) $$\frac{z_{e}}{\rho_{e}-\rho_{0}}\;\exp\left(-\frac{\rho_{e}-\rho_{0}}{\lambda_{\text{D}}}\right)=\frac{\rho_{1}z_{0}}{\rho_{0}}.$$

In the right-hand side of this equation, for convenience, we have expressed the thermal kinetic energy in units of the unscreened potential in the clean channel at a distance *ρ*_0_ from the surface, so *ρ*_1_ is an nondimensional coefficient proportional to *T*. We have also taken into account the finite bare size of the impurities by using *ρ*_*e*_−*ρ*_0_ instead of *ρ*_*e*_ in the potential energy term. From the above equation, *ρ*_*e *_can be obtained with the help of the principal Lambert *W * function as follows:

(3) $$\rho_{e}=\rho_{0}+\lambda_{\text{D}}\;W\left(\frac{\rho_{0}z_{e}}{\lambda_{\text{D}}\rho_{1}z_{0}}\right).$$

Although *W*(*x*) can be easily evaluated by modern computers, it is worthwhile to mention its asymptotes *W*(*x*)≃*x* for *x *≪ 1 and W(x)≃lnx−lnlnx for x≳3. In particular, the first limit means that for small values of *z*_*e*_, it is recovered in the linearity of *ρ*_*e *_with *z*_*e*_ found for the unscreened interaction, with $\rho_{1}^{\prime }=\rho_{0}/(\rho_{1}z_{0})$. In the remaining part of this letter, we shall use the full Equation 3 for the *ρ*_*e*_(*z*_*e*_) functionality.

We may now obtain the fraction *f*_*e *_of impurities that flow, at given *t* and *x* values, near a collision distance from the impurity-dressed wall. For that, we assume that the fluid velocity profile is given by the Poiseuille law, [10]$u(r)\propto r_{e}^{2}-r^{2}$, where *u* is the fluid velocity and *r* the distance to the channel’s axis (see [11] for an explicit discussion supporting that at least for channels of radius ≳10 nm, the flows of water-like liquids driven by hydrostatic pressure are in fact in the Poiseuille regime). Then, *f*_*e *_is given by the fraction of the fluid mass that passes through the outer ring *r*_*e *_−*ρ*_*e *_≤* r *≤* r*_*e*_, i.e., $f_{e}=\int_{r_{e}-\rho_{e}}^{r_{e}}u(r)rdr/\int_{0}^{r_{e}}u(r)rdr$. The result of those integrations is

(4) $$f_{e}={\left[{\left(∥\frac{\rho_{e}}{r_{e}}∥,-,1\right)}^{2},-,1\right]}^{2}.$$

In the considerations leading to Equation 4, we have implicitly taken the concentration of impurities as constant along the radial coordinate *r*. However, in principle, it could be expected that near the walls the electric potential will influence the distance between impurities. To test whether this effect may be of relevance, a Debye-like concentration profile $C_{\text{imp}}(r)=C_{\text{bulk}}(1+z_{e}C^{\prime }e^{-(r-r_{e})/\lambda_{D}})$ was also considered. The corresponding *f*_*e *_is then given by $\int_{r_{e}-\rho_{e}}^{r_{e}}C_{\text{imp}}(r)u(r)rdr/\int_{0}^{r_{e}}C_{\text{imp}}(r)u(r)rdr$, the explicit algebraic result being too cumbersome to be reproduced here. As it will be commented on in detail later in this letter, we have observed that both Equation 4 and the more complicated alternative are able to predict essentially the same filtering performances and time evolutions, and so in the following, we will employ the simpler Equation 4 unless stated otherwise.

The second influence played by *z*_*e *_in our model concerns the probability that an impurity gets actually bound to the inner wall of the channel once it actually is within a collision distance from that wall. We express the probability that a given impurity entering a differential slice of the channel with thickness *dx* gets trapped in that slice as $\Omega_{\text{trap}}=f_{e}\Omega_{\text{trap}}^{\prime }dx$, where $\Omega_{\text{trap}}^{\prime }$ is then a trapping probability per unit length for the impurities flowing near a collision distance from the surface. This $\Omega_{\text{trap}}^{\prime }$ will obviously depend on the chemistry of impurities and active centers of the nanostructure and also on the number density of active centers not yet saturated by existing bindings. The latter indicates that $\Omega_{\text{trap}}^{\prime }$ will grow with *z*_*e*_, and in particular, we may adopt the natural first-order approximation $\Omega_{\text{trap}}^{\prime }=\Omega_{0}+\Omega_{1}z_{e}$ (Ω_0_corresponds then to the value in a conventional non-nanostructured filter and Ω_0 _≪* Ω*_1_*z*_0_).

### Equation for ***∂n(x,t)/∂t***

Let us now build, on the basis of the above relationships, equations for the evolution of the areal density of trapped impurities, *n*, as a function of time *t* and position *x* when an impure fluid flows through the channel due to hydrostatic pressure. We start by considering the differential channel slice going from *x* up to *x* + *dx*, and we write down the expression for *∂n*/*∂t* evaluated at that slice, which may evidently be expressed as Φ_imp_Ω_trap_/(2*Πr*_0_*dx*), where Φ_imp_ is the flow of impurities brought by the incoming fluid (in units of s^−1^; the factor (2*Πr*_0_*dx*)^−1 ^is due to the areal density normalization in the definition of *n*). In its turn, Φ_imp_ can be written as Φ_imp _=* C*_imp_Φ where Φ is the fluid flow and *C*_imp _the incoming number concentration of impurities. Gathering together the previous results in this letter, we get

(5) $$\begin{array}{ll} \;\frac{\partial n(x,t)}{\partial t}= & \frac{C_{\text{imp}}(x,t)\Phi (t)}{2\Pi r_{0}}\left[\Omega_{0},+,\Omega_{1},z_{e},(,n,(,x,,,t,),)\right]\\ & \times {\left[{\left(∥\frac{\rho_{e}(z_{e}(n(x,t)))}{r_{0}-r_{1}n(x,t)}∥-1\right)}^{2}-1\right]}^{2}, \end{array}$$

with the *z*_*e*_(*n*) and *ρ*_*e*_(*z*_*e*_) dependences given by Equations 1 and 3.

### Equations for ***Φ(t)***and ***∂C***_imp_***(x,t)/∂x***

In order to solve the filtration dynamics (i.e., to obtain *n*(*xt*) and *C*_imp_(*xt*)), it is necessary to supplement Equation 5 with formulas for Φ(*t*) and *C*_imp_(*xt*). Regarding the fluid flow, we apply the Poiseuille law for incompressible fluids of viscosity *η* in a cylindrical channel of length *L* and radius *r*_*e*_(*xt*): [10]

(6) $$\Phi (t)=\frac{\Pi P}{8\eta \int_{0}^{L}{\left[r_{0},-,r_{1},n,(,x,,,t,)\right]}^{-4}dx}\;.$$

In this equation, *P* is the pressure difference between both ends of the finite-length channel, which we take constant with time. Note that Φ becomes zero when at some *x*, the *n* value becomes *n*^clog ^≡* r*_0_/*r*_1_, i.e., *r*_*e*_ becomes zero at that location and the channel becomes fully closed by impurities. Note also that Equation 6 reduces in the particular case *r*_0 _≫* r*_1_*n*(*x*,*t*) (which is common in experiments) to $\Phi =\Pi Pr_{0}^{4}/(8\eta L)$.

We construct now the supplementary equation for *C*_imp_(*x*,*t*). For that, we again consider the differential channel slice going from *x* up to *x* + *dx*. The number of impurities that become trapped in its walls during an interval *dt* is (2*Πr*_0_*dx*)(*∂n*/*∂t*)*dt* (the factor 2*Πr*_0_*dx* is again due to the areal normalization in the definition of *n*). The numbers of impurities entering and exiting the slice in the liquid flow are Φ(*t*)*C*_imp_(*x*,*t*)*dt *and Φ(*t*)*C*_imp_(*x* + *dx*,*t*)*dt *respectively. Mass conservation balance therefore gives

(7) $$\frac{\partial C_{\text{imp}}(x,t)}{\partial x}=\frac{-2\Pi r_{0}}{\Phi (t)}\frac{\partial n(x,t)}{\partial t}.$$

**Figure 2:**
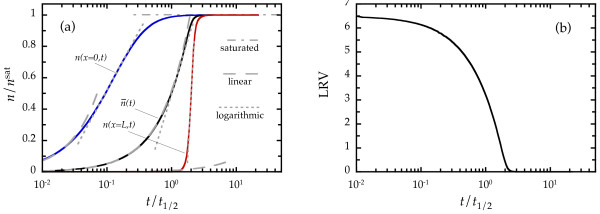
**Time dependence.** (**a**) Results, obtained by integrating Equations 5 to 7, for the time dependence of the areal density of trapped impurities (continuous lines) at the entrance of the channel *n*(*x *= 0,*t*) and at its exit point *n*(*x *=* L*,*t*), and also the global average areal density of trapped impurities $\bar{n}(t)=L^{-1}\int_{0}^{L}n(x,t)\text{d}x$. The areal density axis is normalized by the saturation value *n*^sat^. The time axis is normalized by the half-saturation time, defined by $\bar{n}(t_{1/2})=n^{\text{sat}}/2$. The parameter values used are as follows (see main text for details): *ρ*_0 _= 13 nm, *ρ*_1 _= 0*.*11, *λ*_D _= 5*.*1 nm, $n^{\text{sat}}=1.5\times 10^{15}/{\text{m}}^{2}=0.25/\rho_{0}^{2}$, *r*_0 _= 500 nm, $r_{1}=8.8\times 10^{-24},{\text{m}}^{3}=4\rho_{0}^{3}$, Ω_0 _= 0, Ω_1_*z*_0 _= 1*.*2 × 10^5^/m, *L *= 7*.*25 mm, *P *= 3×10^5^ Pa, *η *= 10^−3 ^Pa·s, and $C_{\text{imp}}(x=0,t)=10^{10}/{\text{m}}^{3}$. We also show the linear, logarithmic, and saturated behaviors (as dashed, dotted, and dot-dashed lines respectively). (**b**) Time dependence of the logarithmic removal value (LRV), calculated using the same parameter values as in Figure 2a.

Notice that Equations 5 to 7 are coupled to each other. In fact, they form now a closed set that can be numerically integrated by providing the specific values for the characteristics of the filter, for any given pressure difference *P* and incoming impurity concentration *C*_imp_(0,*t*). In what follows, for simplicity, we will always consider for the latter a constant value *C*_0_. The computation to numerically integrate Equations 5 to 7 is relatively lightweight (e.g., calculating our Figure 2 took about 15 min in a current personal computer that considered 2 × 10^4 ^finite-element *x*-slices).

## Discussion of the results obtained by integrating the model equations

### Numerical integration and comparison with some existing partial measurements

We show in Figure 2 an example of the results obtained by numerically integrating Equations 5 to 7 using some representative values for the parameters involved (and always in the case of constant *P* and *C*_imp_, and starting from a clean initial state *n*(*xt *= 0)=0). In particular, we have chosen parameter values that reproduce the case of channels coated with Y_2_O_3_ nanopowders as measured in [5] (they are essentially valid also for the quite similar case of channels with ZrO_2_ nanocoating reported by the same group in [6]). In these filters, the channels have a typical value of the nominal radius *r*_0 _= 500 nm and length *L *= 7*.*25 mm. They were shown [5] to efficaciously retain MS2 viruses (of radius *ρ*_0 _= 13 nm) carried by water with NaCl as background electrolyte and a conductivity of 400*μ*S/cm (corresponding then to *λ*_D_≃5*.*1 nm) feed at a pressure *P *= 3 bar. The incoming impurity number concentration was $C_{\text{imp}}(x=0,t)=10^{10}/{\text{m}}^{3}$. For the saturation areal density *n*^sat^, we will estimate, based on figure nine of [5], a quite conservative value *n*^sat ^= 1*.*5 × 10^15^/m^2^, corresponding to $0.25/\rho_{0}^{2}$. For the parameter *r*_1_, we will use the value $4\rho_{0}^{3}$, also consequent in the order of magnitude with figure nine of [5]. These numbers imply that at saturation (*n *=* n*^sat^), the effective radius of the channel is $r_{e}^{\text{sat}}=487$ nm. Note that this value is rather close to the clean-state value of 500 nm, and then it would correspond to an increase of the hydrodynamic resistance of only about 10% (unfortunately, the nanocoatings in [5,6] seem to be washed out before they can be fully saturated; however, other nanocoated filters [4,7,8] have been shown to have hydrodynamic resistance only moderately increased at saturation, what is indeed an advantage of paramount importance for applications). We will also assume a null $\Omega_{\text{trap}}^{\prime }$ value at the saturated state, i.e., Ω_0 _= 0 (so that we neglect conventional filtration mechanisms and focus on the effects of nanostructuring alone). In order to proceed with the numerical calculation of Equations 5 to 7, only two parameters remain to be given estimated values: Ω_1_*z*_0_(Ω_1_ and *z*_0_ do not appear separately in Equations 5 to 7) and *ρ*_1_(or equivalently, via Equation 3, the effective impurity radius in the clean state of the channel, $\rho_{\text{e}}^{\text{clean}}$). We have found that the values Ω_1_*z*_0 _= 1*.*2 × 10^5^/m and *ρ*_1 _= 0*.*11 produce results in reasonable agreement with the available experimental information, as we discuss below. The value *ρ*_1 _= 0*.*11 corresponds to $\rho_{\text{e}}^{\text{clean}}=33$ nm, or *ρ*_0_ + 4*λ*_D_.

Figure 2a presents the results corresponding to integrating Equations 5 to 7 using these parameter values, for the areal density of trapped impurities at the entrance of the channel *n*(*x *= 0,*t*) and at its exit point *n*(*x *=* Lt*) and also for the global average areal density of trapped impurities $\bar{n}(t)=L^{-1}\int_{0}^{L}n(x,t)dx$. Figure 2b presents the corresponding logarithmic removal value (LRV), calculated as $-\log_{10}[C_{\text{imp}}(x=L,t)/C_{\text{imp}}(x=0,t)]$. Note that in Figure 2a,b, the time axis is logarithmic and that for convenience, it was normalized by the time *t*_1/2_ defined by the condition $\bar{n}(t_{1/2})=n^{\text{sat}}/2$ (half-saturation time). The agreement of these numerical results with the measured filtration performance reported in [5,6] is fairly good. In particular, we obtain an initial LRV of 6.5 log, equal to the LRV measured in [5,6] when the actual filters (composed by a macroscopic array of microchannels) were challenged with only about 1 L of water (the authors of [5,6] estimate that such volume carries a total amount of impurities that is orders of magnitude smaller than the total available binding centers in their filter, so the measurement is expected to correspond to almost clean channels, as in fact seems to be confirmed by microscopy images [5]). The calculated LRV is of 4 log at *t*/*t*_1/2_≃0*.*7, which is also in fair agreement with the observation of a 4 log filtration in [5,6] after passing through the macroscopic filter approximately from 200 to 1,000 L, depending on the measurement. However, obviously, a more stringent determination of the parameter values, and in general of the degree of validity of our equations, would need more precise and detailed data. Unfortunately, to our knowledge, no measurements exist for the time evolution of the filtering efficiency of channels with nanostructured walls with a *t*-density and precision sufficient for a fully unambiguous quantitative comparison with the corresponding results of our equations; in fact, one of the main motivations of the present Nano Idea Letter is to propose (see our conclusions) that such measurements should be made, in order to further clarify the mechanism behind the enhanced impurity trapping capability of the channels with nanostructured inner walls.

As a further test, we have repeated the same numerical integration as in Figure 2a,b but considering a radial impurity concentration profile $C_{\text{imp}}(r)\propto 1+z_{e}C^{\prime }e^{-(r-r_{e})/\lambda_{D}}$, instead of a constant one as in Equation 4. We have obtained very similar results, provided that the parameter Ω_1_*z*_0_ is conveniently varied: In particular, we observed that the filtration dynamics results obtained using Equation 4 and any given value *γ* for Ω_1_*z*_0 _can be reproduced using the above Debye-like profile if employing for Ω_1_*z*_0_ a new value (specifically, the new value can be estimated, by comparing the initial filtration performance, as $\gamma I_{r_{0}-\rho_{e}^{\text{clean}}}^{1}I_{0}^{C_{\text{imp}}(r)}/I_{0}^{1}I_{r_{0}-\rho_{e}^{\text{clean}}}^{C_{\text{imp}}(r)}$, where $I_{x}^{y(x)}=\int_{x}^{r_{e}}y(x)u(x)dx$ ; for instance, taking $z_{0}C^{\prime }=200$, which probably is a fair first approximation for the measurements in [5-8], the parameter values used in Figure 2 correspond to 3*.*2 × 10^4^/m as equivalent Ω_1_*z*_0 _value when using the Debye approach). These results indicate then that, as it could be expected, the wall charge effect on the radial gradient of the concentration may be safely summarized, for our present purposes, as one of the factors influencing the value of the trapping probability coefficient Ω_1_*z*_0_ to be used when applying Equation 4.

### Linear, logarithmic, and saturated approximations

In Figure 2a, it is possible to identify in our results for the areal density of trapped impurities some *t*-ranges in which the *t*-dependence is relatively simple: (1) The initial time behavior is an approximately linear *n*(*t*) growth; (2) in the intermediate regime, the growth of *n*(*t*) becomes approximately logarithmic; and (3) at sufficiently large *t* values, the saturation limit is reached, in which *n* approaches a value *n*^sat^ at a slow pace. These regimes are easily seen in Figure 2a for *n*(*x *= 0,*t*), *n*(*x *=* L*,*t*), and $\bar{n}(t)$, albeit in each case they are located at different *t*/*t*_1/2 _ranges. The figure also evidences that it is possible for the linear and logarithmic *t*-ranges to overlap each other (the case of $\bar{n}(t)$ with the parameter values used in Figure 2).

In the case of a very short cylindrical channel (so that all *x*-derivatives may be neglected), it is possible to find analytical expressions for the *n*(*t*) evolution in the linear and logarithmic regions: For the linear regime, by just introducing in Equation 5 the condition *t *≃ 0, we find:

(8) $$n(t)≃A_{\text{lin}}t$$

with

(9) $$\begin{array}{ll} \;A_{\text{lin}}= & \frac{(\Omega_{0}+\Omega_{1}z_{0})C_{\text{imp}}P}{16\eta Lr_{0}}\\ & \times {\left[{\left(\rho_{0}-r_{0}+\lambda_{\text{D}}\;W\left(\frac{\rho_{0}}{\lambda_{\text{D}}\rho_{1}}\right)\right)}^{2}-r_{0}^{2}\right]}^{2}. \end{array}$$

The logarithmic regime can be found by using the condition *n *≃* n*^sat^/2:

(10) $$n(t)≃\frac{n^{\text{sat}}}{2}+A_{\text{log}}\ln\frac{t}{t_{1/2}}$$

with

(11) $$\begin{array}{ll} \;A_{\text{log}}= & \frac{(2\Omega_{0}+\Omega_{1}z_{0})\;t_{1/2}C_{\text{imp}}P}{512\eta L\;r_{0}}\\ & \times {\left[{\left(2\rho_{0}-2r_{0}+\lambda_{\text{D}}\;W\left(\frac{\rho_{0}}{\lambda_{\text{D}}\rho_{1}}\right)+r_{1}n^{\text{sat}}\right)}^{2}-{\left(2r_{0}-r_{1}n^{\text{sat}}\right)}^{2}\;\right]}^{2}\;. \end{array}$$

In obtaining the above Equations 8 to 11, we have assumed that *n*(0) = 0 and that *ρ*_*e *_<* r*_*e*_ at *t *= 0 or *t*_1/2_.

## Conclusions and proposals for future work

This letter has proposed a model for the main generic features of the channels with nanostructured inner walls with respect to trapping and accumulation of impurities carried by fluids. This includes, e.g., their capability to clean the fluid from impurities of a size much smaller than the channels’ nominal radius, with comparatively small resistance to flow (much smaller than in conventional channels with a radius as small as the impurities). The model attributes the enhanced filtration capability to the long-range attraction exerted by the exposed charges in the nanostructured walls and also to their binding capability once the impurities actually collide with them. Both features were quantitatively accounted for by means of a phenomenological ‘effective-charge density’ of the nanostructured wall. The model also predicts the time evolution of the trapped impurity concentration and of the filtering capability, including three successive regimes: a linear regime, a logarithmic regime, and the saturated limit.

We believe that our equations could make possible some valuable future work, of which two specific matters seem to us more compelling: First, it would be interesting to check at the quantitative level the agreement with experiments of the time evolutions predicted above. For that, we propose to perform time-dependent measurements made in controlled flow setups. We have chosen in our equations flow constraints which seem appropriate for this purpose; however, the model can be tested with different setups by just numerically integrating Equation 5 under the corresponding experimental constraints. One of the prime purposes of this letter is in fact to suggest such measurements.

A second interesting future work (already in progress in our research group) is the design of optimal geometries for the combinations of channels forming filters. Our model opens this possibility because of the explicit use of the Poiseuille relations that allow the calculation of the resistance to flow of complex associations of those channels, in series and/or parallel. The effective diffusivity and tortuosity of the pathways’ network are also accounted for by these equivalent-circuit analyses.

## Competing interests

The author declares that he has no competing interests.
